# Pyrosequencing of the bacteria associated with *Platygyra carnosus* corals with skeletal growth anomalies reveals differences in bacterial community composition in apparently healthy and diseased tissues

**DOI:** 10.3389/fmicb.2015.01142

**Published:** 2015-10-20

**Authors:** Jenny C. Y. Ng, Yuki Chan, Hein M. Tun, Frederick C. C. Leung, Paul K. S. Shin, Jill M. Y. Chiu

**Affiliations:** ^1^Department of Biology, Hong Kong Baptist UniversityHong Kong, Hong Kong; ^2^Oral Biosciences, Faculty of Dentistry, The University of Hong KongHong Kong, Hong Kong; ^3^School of Applied Sciences, Institute for Applied Ecology New Zealand, Auckland University of TechnologyAuckland, New Zealand; ^4^School of Biological Sciences, The University of Hong KongHong Kong, Hong Kong; ^5^Department of Animal Science, University of ManitobaWinnipeg, MB, Canada; ^6^Department of Biology and Chemistry, City University of Hong KongHong Kong, Hong Kong; ^7^State Key Laboratory in Marine PollutionHong Kong, Hong Kong

**Keywords:** *Platygyra carnosus*, coral disease, skeletal growth anomalies, microbial communities, metagenomics

## Abstract

Corals are rapidly declining globally due to coral diseases. Skeletal growth anomalies (SGA) or “coral tumors” are a group of coral diseases that affect coral reefs worldwide, including Hong Kong waters in the Indo-Pacific region. To better understand how bacterial communities may vary in corals with SGA, for the first time, we examined the bacterial composition associated with the apparently healthy and the diseased tissues of SGA-affected *Platgyra carnosus* using 16S ribosomal rRNA gene pyrosequencing. Taxonomic analysis revealed Proteobacteria, Bacteroidetes, Cyanobacteria, and Actinobacteria as the main phyla in both the apparently healthy and the diseased tissues. A significant difference in the bacterial community composition was observed between the two conditions at the OTU level. Diseased tissues were associated with higher abundances of Acidobacteria and Gemmatimonadetes, and a lower abundance of Spirochaetes. Several OTUs belonging to Rhodobacteraceae, Rhizobiales, Gammaproteobacteria, and Cytophaga-Flavobacterium-Bacteroidetes (CFB) were strongly associated with the diseased tissues. These groups of bacteria may contain potential pathogens involved with the development of SGA or opportunistic secondary or tertiary colonizers that proliferated upon the health-compromised coral host. We suggest that these bacterial groups to be further studied based on inoculation experiments and testing of Koch's postulates in efforts to understand the etiology and progression of SGA.

## Introduction

In the past two decades, coral reefs have been rapidly declining due to environmental impacts, such as infectious diseases (Harvell et al., 2007), bleaching (Baker et al., 2008), fishing overexploitation (Willis et al., 2004), predator outbreaks (Kayal et al., 2012), and global climate change (Hoegh-Guldberg et al., 2007). The reduction of reef-building corals contributes to the overall degradation of marine ecosystems, including the decline in abundance and diversity of reef fish (Jones et al., 2004; Paddak et al., 2009). Coral diseases are known as one of the most significant contributors to the decline of corals worldwide (Miller and Richardson, 2011), resulting in the total loss of coral cover in some regions in the Indo-Pacific and Australia (Bruno and Selig, 2007; Sweatman et al., 2011).

Skeletal growth anomalies (SGA) or “coral tumors” are a group of coral diseases affecting the major reefs of the Indo-Pacific (Chiu et al., 2012; Tavakoli-Kolour et al., 2015), Australia (Haapkylä et al., 2010), Hawaii (Stimson, 2011), Costa Rica (Gateño et al., 2003), and the Philippines (Kaczmarsky and Richardson, 2011). SGA are abnormal tissue growths which result in raised and enlarged areas that differ markedly in morphology from the adjacent healthy tissues (Breitbart et al., 2005). Corals with SGA are associated with rapid growth (Gateño et al., 2003), a reduced number of polyps and symbiotic zooxanthellae, finer skeletal structures, and a decline in fecundity and calcification rates (Stimson, 2011). Although the etiology of SGA is unknown, studies have shown that the disease is linked to environmental factors, such as climate change (Rosenberg and Ben-Haim, 2002), sea surface temperature, and human population size (Aeby et al., 2011). For instance, the increase in sea surface temperature is often attributed for inducing physiological stress, which allows for transmission of pathogenic agents in compromised corals (Rosenberg and Ben-Haim, 2002).

Several studies have highlighted the role of bacteria in coral diseases (Sunagawa et al., 2010; Chiu et al., 2012; Godwin et al., 2012). Corals harbor a large diversity of bacteria, many of which have been shown to be beneficial by promoting coral health, defense, and nitrogen fixation (Richie, 2006; Chimetto et al., 2008). It has been suggested that bacteria contribute to the corals' ability to adapt to changes in the environment (Reshef et al., 2006), which may result in disease when under environmental stress (Rosenberg et al., 2007). Coral diseases have been shown to cause changes to the structure, diversity, and abundance of bacterial communities (Harvell et al., 2007). Meta-analysis on coral-associated bacterial communities revealed that corals with diseases generally have more *Rhodobacter* and Cyanobacteria sequences (Mouchka et al., 2010; Miller and Richardson, 2011). Members of the *Vibrio* have been extensively studied due to their identification as etiological agents in a number of coral diseases (Luna et al., 2010; Sweet et al., 2014). Other microorganisms targeted as potential pathogens include *Thalassomonas loyana* for white plague disease (Thompson et al., 2006), *Cytophaga* sp. for black band disease (Cooney et al., 2002; Miller and Richardson, 2011), and *Serratia marcescens* for white box disease (Harvell et al., 2007). While no clear evidence of proliferation of bacterial, viral and fungal pathogens has been observed in corals with SGA yet, a faster growth rate of bacterial cells has been observed in corals with SGA (Breitbart et al., 2005) and SGA appeared to be transmissible between colonies (Kaczmarsky and Richardson, 2007).

Very little is known about the bacterial communities of corals with SGA in the Indo-Pacific. SGA have been documented to affect 26 scleractinian coral species in the region (Sutherland et al., 2004), including *Platygyra carnosus* (Chiu et al., 2012), *P. daedalea* (Tavakoli-Kolour et al., 2015), *P. pini*, and *P. sinensis* (Sutherland et al., 2004). In Southern China, *P. carnosus* is a dominant scleractinian coral, forming the major structural framework of coral communities (Veron, 2000). A recent survey of Hong Kong reef-building corals indicated that 63.5% of *P. carnosus* colonies have developed SGA (Chiu et al., 2012), which highlights the importance of deepening our understanding of the bacterial communities associated with SGA.

We recently observed no difference between the bacterial communities associated with remote healthy and SGA-affected *P. carnosus* corals using the culture-dependent method (Chiu et al., 2012). Except for our previous work conducted in Hong Kong, no other previous microbiological work has pursued to compare healthy and SGA-affected coral colonies. In addition, no studies are available on the diversity of the coral bacterial community for *P. carnosus*. Thus, the bacterial composition involved with SGA in *P. carnosus* remains largely unknown. To gain better understanding of how bacterial communities associated with corals may vary within colonies affected by SGA, this paper presents the first culture-independent study to provide detailed characterization of the bacterial community composition associated with the apparently healthy and the diseased tissues of SGA-affected *P. carnosus* colonies. Bacterial communities were detected using 454 pyrosequencing of the 16S ribosomal rRNA genes, which have been previously employed by various studies to investigate coral-associated communities (Cárdenas et al., 2011; Cróquer et al., 2012; Godwin et al., 2012).

## Materials and methods

### Site description and sample collection

The sampling of *Platygyra carnosus* colonies for this study was authorized by the Agriculture, Fisheries and Conservation Department of Hong Kong (Permit AF GE MPA 01/5/2 pt11).

**Figure 1:**
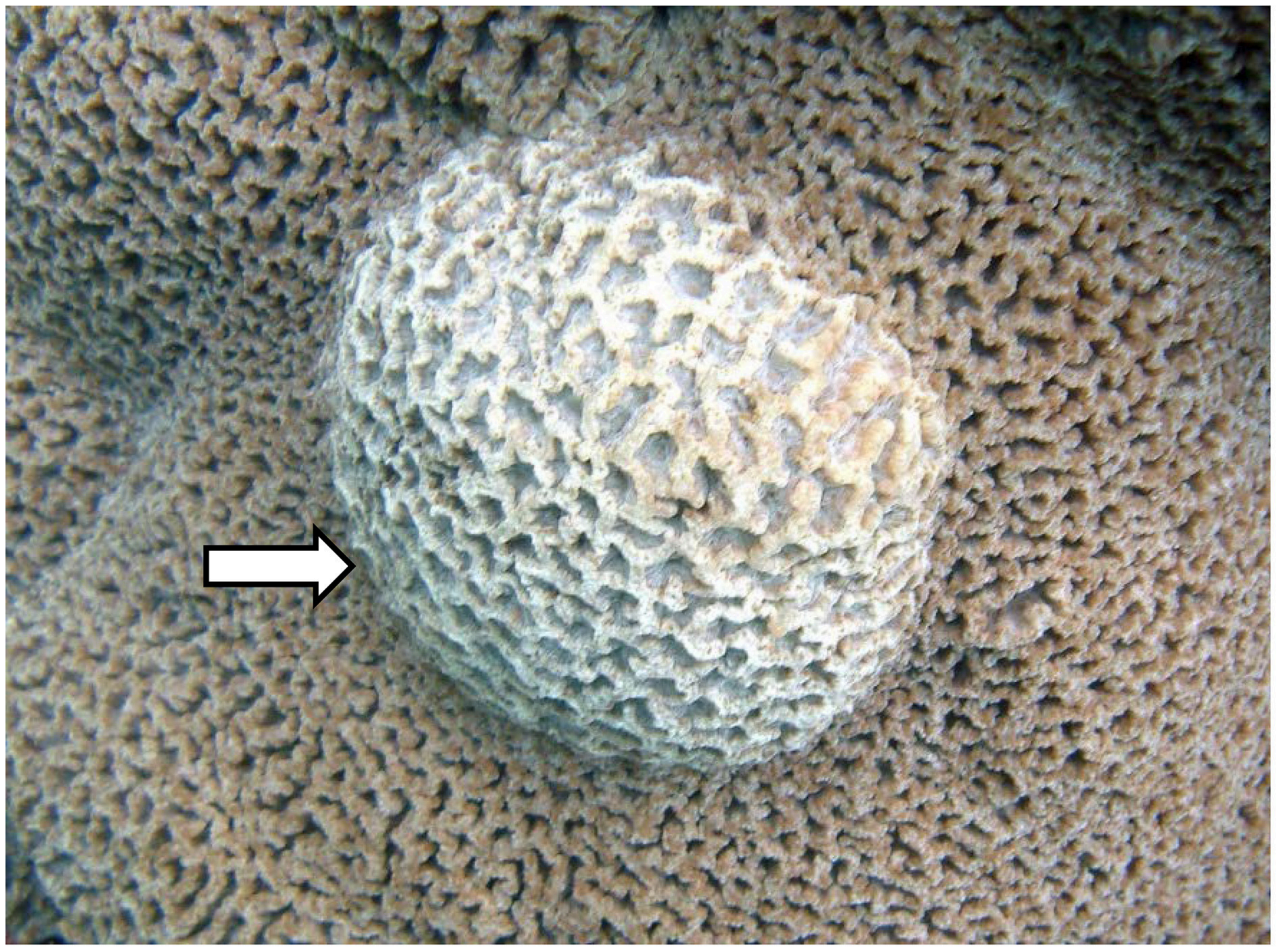
**Picture of *Platgyra carnosus* skeletal growth anomaly (SGA) as indicated by an arrow and normal tissue on the remaining areas**.

Samples of *Platygyra carnosus* exhibiting signs of SGA were collected on May 30, 2011 at Hoi Ha Wan Marine Park (22° 28.896′ N, 114° 19.996′ E) off the northwest side of Mo Chau (Moon Island) in Hong Kong. Samples were collected from the apparently healthy and the diseased polyps of four replicate SGA-affected *P. carnosus* colonies. Diseased samples were collected from tissues that displayed abnormal raised growths (Figure 1), and apparently healthy samples were collected from tissues with no visible signs of disease. Apparently healthy and diseased samples were at least 20 cm apart from each other. A modified air-driven industrial drill was used to drive coral cores of 12 mm diameter and 30–40 mm thick. All samples were washed twice by submersion in autoclaved and membrane-filtered (pore size 0.22 μm) seawater to remove loosely attached microorganisms. The core holes were subsequently filled with epoxy paste (PC11; Protective Coating Co., Allentown, PA, USA) to prevent infection. All samples were immediately transported from the collection site to the laboratory in extraction buffer (100 mM Tris–HCl, 100 mM Na_2_ · EDTA, 100 mM NaH_2_PO_4_, 1.5 M NaCl, and 1% CTAB) in sterile conical tubes in an icebox.

### Extraction of genomic DNA, PCR amplification and pyrosequencing

Coral samples were crushed using a mortar and pestle and vortexed for 3 min at maximum speed. Bacterial genomic DNA was extracted with phenol-chloroform-isoamyl alcohol (Liu et al., 1997) and purified with ammonium acetate precipitation (Miller et al., 1999). PCR amplification of 16S rRNA genes was performed using the FastStart High Fidelity PCR system (Roche Molecular Diagnostics, Branchburg, NJ, USA) with primers 341F (5′- ACTCCTACGGGAGGCAGCAG-3′) and 926R (5′- CCGTCAATTCMTTTGAGTTT-3′) targeting the hypervariable V3–V5 region. Both forward and reverse primers were fused to Roche Fusing primers A and B, with each Fusion primer A having a unique 10 base DNA barcode specific for each sample. PCR products were verified for quality, size, and quantity by gel electrophoresis and PicoGreen Assay. Equimolar quantities of PCR products were pooled and separated by 1% agarose gel electrophoresis. DNA corresponding to the amplified 16S rRNA gene was excised from the gels and purified using PureLink® Quick Gel Extraction Kit (Invitrogen Life Technologies, Hong Kong, China). Amplicon libraries for all samples were subjected to pyrosequencing using a bench-top 454 GS Junior (454 Life Sciences-a Roche Company, Branford, CT, USA). All sequences obtained were submitted into the National Centre for Biotechnology Information (NCBI) database (accession number SRP028284).

### Bioinformatics and statistical analyses

Raw pyrosequence data were separated into three files (.fasta, .qual, and .txt) using sff software tools from Roche/454. Subsequent data were processed and analyzed using the Quantitative Insights Into Microbial Ecology (QIIME) pipeline (Caporaso et al., 2010b). Sequences with a mean quality score lower than 25, length of < 200 bp or >900 bp, missing primer sequences, contained ambiguous bases, contained homopolymer runs exceeding eight nucleotides or uncorrectable barcodes were removed from further analysis. Demultiplexing was performed using sample-specific barcode sequences. Denoising of sequences was performed using DENOISER v. 0.9.1 (Reeder and Knight, 2010; Quince et al., 2011), as implemented in the QIIME platform. Chimeric sequences were removed using Chimera Slayer (Hass et al., 2011). The remaining good quality sequences were rarefied and assigned into Operational Taxonomic Units (OTUs) at a threshold of 97% pair-wise nucleotide sequence identity using UCLUST (Edgar, 2010). Sequences were subjected to BLAST searches at NCBI to determine their closest relatives in the GenBank nt database (http://blast.ncbi.nlm.nih.gov/Blast.cgi). Representative sequences at the cluster centroid for each OTU were chosen and classified to genus level by the Ribosomal Database Project (RDP) Classifier at a confidence threshold of 80% (Wang et al., 2007). The OTUs were aligned using PyNAST (Caporaso et al., 2010a) with a minimum alignment length of 150 bp and a minimum identity at 75%. PH LANE mask was performed to screen away hypervariable regions. An approximately-maximum-likelihood phylogenetic tree was constructed using FastTree (Price et al., 2010) with Kimura's 2-parameter model. Alpha diversity analysis was performed by computing rarefaction curves, diversity estimates (Shannon-Weaver index) (Magurran, 1988) and nonparametric richness estimates (Chao1) (Lee and Chao, 1994). Similarity in community composition between the apparently healthy and diseased tissues of SGA-affected colonies was compared with random even subsampling and 1000 Monte Carlo iterations using unweighted and weighted UniFrac distances visualized in Principal Coordinates Analysis (PCoA) (Lozupone et al., 2006). Similarity Percentage (SIMPER) analysis was performed to identify the taxa that contributed to most of the variance among samples (Clarke, 1993). Wilcoxon-Mann-Whitney rank sum test and Metastats test (White et al., 2009) were performed to determine the differences between the apparently healthy and diseased tissue samples for individual taxa. Analysis of similarity (ANOSIM) and Permutational Multivariate Analysis of Variance (PERMANOVA) based on the Bray-Curtis dissimilarity index (Bray and Curtis, 1957) were performed to test for the overall community differences in composition and abundance of all taxa between the apparently healthy and diseased samples with 9999 permutations. All statistical tests, unless specified, were performed in R (version 2.15.2, R Core Team, 2012).

## Results

### Diversity of bacteria associated with the apparently healthy and the diseased tissues of *P. carnosus*

**Figure 2:**
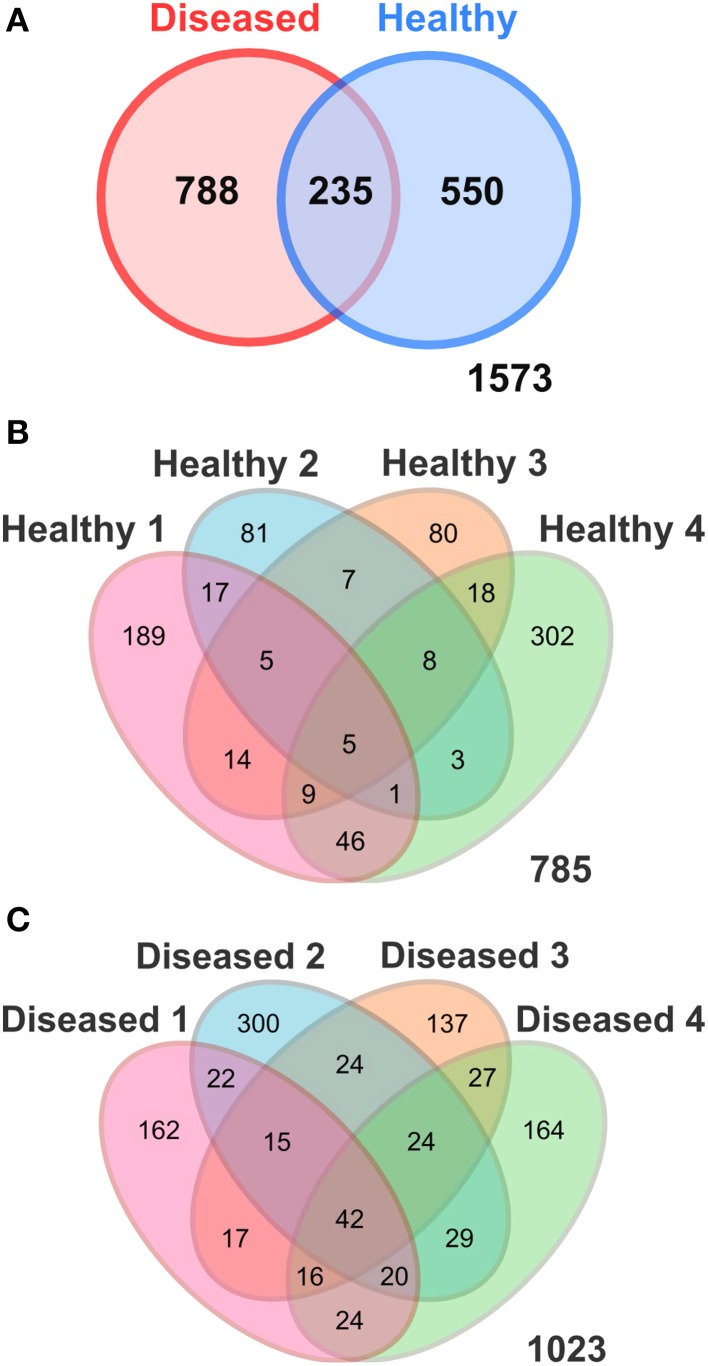
**Venn Diagrams illustrating the number of shared OTUs (97% sequence similarity) (A) between the apparently healthy and diseased *P. carnosus* tissues; and among the replicates of (B) the apparently healthy and (C) the diseased tissues detected in *P. carnosus* by pyrosequencing**. Healthy, apparently healthy tissues of SGA affected *P. carnosus* colonies; Diseased, diseased tissues of SGA affected colonies.

A total of 108,015 good quality sequences, subdivided into 45,077 sequences for the apparently healthy tissues, and 62,938 sequences for the diseased tissues, were obtained from SGA-affected colonies. Diversity analysis and number of observed Operational Taxonomic Units at 97% sequence similarity were performed by averaging four random subsamples of 1802 sequences each, corresponding to the lowest number of sequences obtained from one of the coral tissue samples. Average values after even subsampling were 14,416 sequences, including 7208 sequences from both the apparently healthy and diseased tissues (Table 1). Sequences clustered into a total of 1573 OTUs, of which 550 were exclusively associated with the apparently healthy tissues, 788 were exclusively associated with the diseased tissues, and 235 were shared by the two respective groups (Table 1, Figure 2A).

**Table 1:** **Summary of the bacterial diversity found by pyrosequencing of the apparently healthy and the diseased tissues of SGA-affected *P. carnosus* colonies**.

	**Diseased**	**Healthy**
No. of assigned phyla	17	17
No. of assigned classes	38	31
No. of assigned orders	67	56
No. of assigned families	96	83
Count of OTUs	1023	785
Count of unique OTUs	467	344
Count of OTUs exclusive to each group	788	550
Count of exclusive unique OTUs	410	282
No. of OTUs shared by the two groups	235	
Count of sequence tags	7208	7208
Phylogenetic diversity index ± SD	27.1 ± 2.4	21.0 ± 4.2
Richness estimate (Chao1) ± SD	579.8 ± 56.3	349.4 ± 107
Diversity estimate (Shannon-Weaver) ± SD	6.6 ± 0.4	5.9 ± 0.4

Count of OTUs is the number of operational taxonomic units observed. Count of unique OTUs is the number of OTUs containing only one sequence tag. Count of sequence tags is the total number of sequences obtained. Numbers of phyla, classes, orders and families are based on taxonomic classification by the Ribosomal Database Project (RDP) Classifier. Phylogentic diversity index is a measure of biodiversity based on their phylogenetic divergence. Chao 1 is an estimator of the minimum richness. Shannon-Weaver Index combines estimates of richness (total number of OTUs) and evenness (relative abundance). Healthy, apparently healthy tissues of SGA affected P. carnosus colonies; Diseased, diseased tissues of SGA affected colonies.

**Figure 3:**
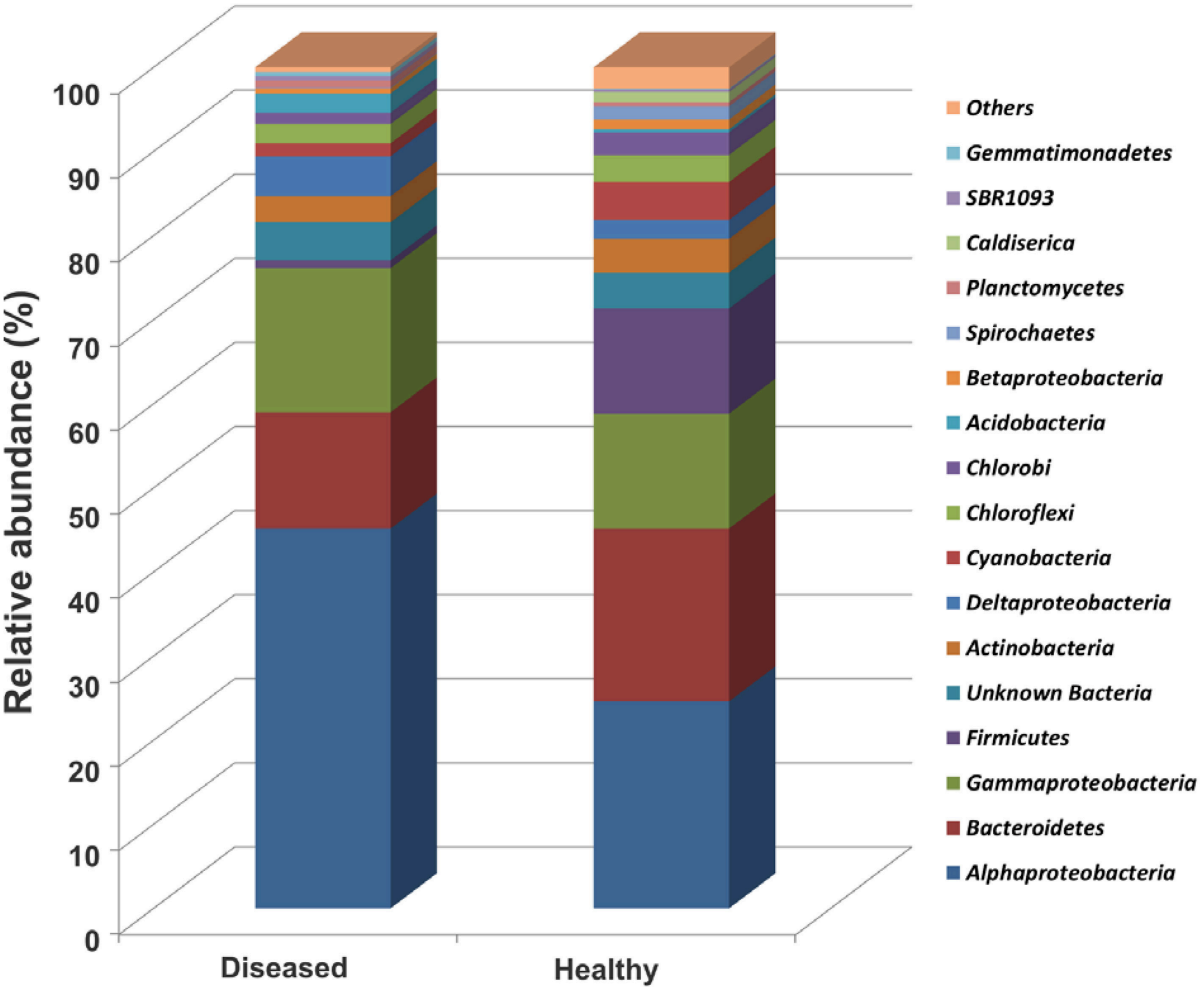
**Relative abundance (%) of major bacterial phyla (except Proteobacteria into class) in sequenced OTUs from the apparently healthy and the diseased tissue samples**. Healthy, apparently healthy tissues of SGA affected *P. carnosus* colonies; Diseased, diseased tissues of SGA affected colonies. Stacked bars were calculated from the mean of relative sequence abundance belonging to each phylum in each sample condition.

Taxonomic classification of the 16S rRNA gene sequences using the RDP classifier showed that 99.95% of total sequences belonged to the domain Bacteria, 0.01% to Archaea, and 0.03% to others. Only bacterial sequences were considered in this study. Sequences were classified into 17 phyla, 31 classes, 56 orders, and 83 families for the apparently healthy tissues, and 17 phyla, 38 classes, 67 orders, and 96 families for the diseased tissues (Table 1). A high alpha-diversity (Shannon-Weaver's Index) and richness estimate (Chao1) were observed in both the diseased tissues (mean and standard error of 6.6 ± 0.4 and 579.8 ± 56.3, respectively) and the apparently healthy tissues (5.9 ± 0.4 and 349.4 ± 107, respectively) of SGA-affected colonies (Table 1). The bacterial communities associated with the apparently healthy tissues was dominated by members of the Alphaproteobacteria (25.0%), followed by Bacteroidetes (20.9%), Gammaproteobacteria (13.9%), Firmicutes (12.7%), Cyanobacteria (4.6%), and Actinobacteria (4.1%) (Figure 3, Figure S1). The phylum Proteobacteria represented 42.4% of all analyzed sequences obtained from the apparently healthy tissues and 4.3% were unclassifiable. Diseased tissues exhibited high diversity and was also dominated by members of the Alphaproteobacteria (45.1%), followed by Gammaproteobacteria (17.1%), Bacteroidetes (13.8%), Deltaproteobacteria (4.7%), Actinobacteria (3.1%), Acidobacteria (2.3%), Chloroflexi (2.3%), and Cyanobacteria (1.5%) (Figure 3, Figure S1). The phylum Proteobacteria represented 67.6% of the total number of sequences obtained from the diseased tissues. Firmicutes comprised less than 1% of all the sequences. Of the analyzed sequences from diseased samples, 4.5% were unclassifiable at the phylum level.

### Comparison of bacterial communities associated with the apparently healthy and the diseased tissues

Alpha diversity estimates and rarefaction curves were performed by subsampling to an even number of sequences. The Chao1, Shannon-Weaver, and Phylogenetic diversity indices were comparable across samples indicating that the bacterial community richness and diversity at the selected number of sequences is not different between the apparently healthy and the diseased tissues of SGA-affected colonies (Table 1).

**Figure 4:**
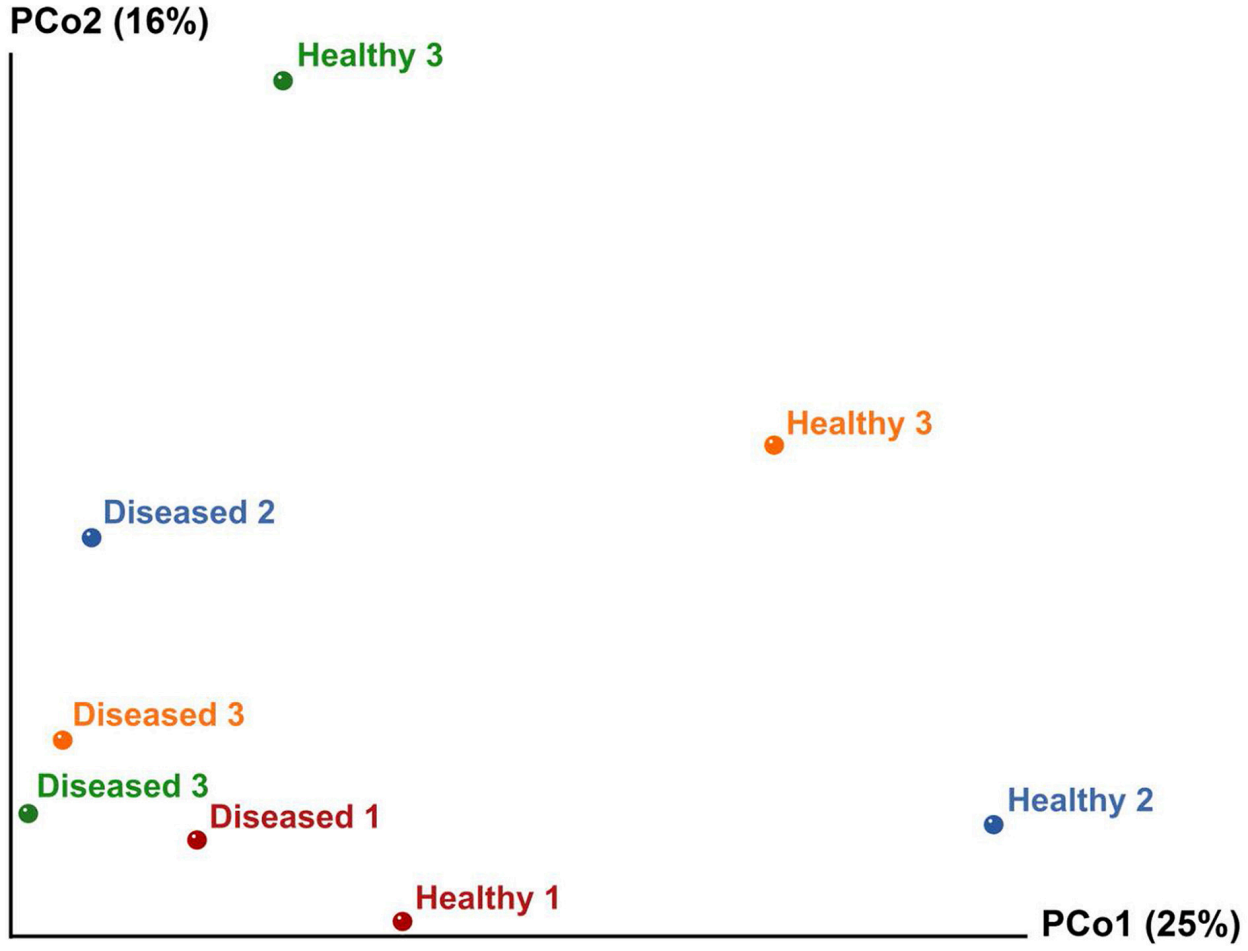
**Principal Coordinates Analysis (PCoA) of the apparently healthy tissues and diseased tissues of tumor affected *P. carnosus* coral based on unweighted UniFrac matrices (weighted data not shown due to similar results)**. Analysis showed clustering of diseased tissues from apparently healthy tissues, suggesting that the presence or absence of certain bacterial lineages may be critical for coral's health.

Multivariate ANOSIM and PERMANOVA analysis based on the Bray-Curtis dissimilarity index revealed clear differences between the two conditions at the OTU (equivalent to genus-to-species) level (one-way ANOSIM, *R* = 0.4688, *p* = 0.0286; PERMANOVA, *F* = 1.879, *p* = 0.028, permutation 9999). This result is supported by UniFrac measures visualized in Principal Coordinates Analysis, which indicates that the bacterial communities generally divided into two groups (Figure 4). However, we observed variable distances in the bacterial communities between replicates of the two conditions that a natural variation between colonies existed (Figure 4). In particular, a higher degree of variation was observed among the bacterial communities of the apparently healthy tissues than the diseased tissues as indicated by the closer clustering of diseased samples in Principal Coordinates Analysis (Figure 4). These results corroborate the Venn Diagrams constructed on the apparently healthy tissues and the diseased tissues (Figures 2B,C), which indicates that there were a low number of OTUs shared by the coral colonies in each condition.

### Differentially abundant bacterial taxa in the apparently healthy vs. the diseased coral tissues

Wilcoxon rank-sum test and Metastats test identified a number of bacterial taxa that were differentially abundant between the apparently healthy and the diseased tissues (Table 2). SIMPER analysis was performed to determine which taxa contributed most to the differences between the two conditions. Analysis at phylum level indicated that Proteobacteria contributed most of the differences in the relative abundance between the apparently healthy and diseased tissues (37.6%), followed by Firmicutes (16.7%), Bacteroidetes (13.4%), Cyanobacteria (5.1%), and Actinobacteria (4.8%) (Table 2). Comparison of the apparently healthy and the diseased tissues showed a higher abundance of bacteria belonging to the phyla Acidobacteria and Gemmatimonadetes, and a lower abundance of bacteria belonging to Spirochaetes in the diseased samples (*p* < 0.05, both Wilcoxon rank-sum test and Metastats test, Table 2). SIMPER analysis at family level indicated that Rhodobacteraceae contributed to most of the differences in the relative abundance between the two conditions (14.6%), followed by Flammeovirgaceae (6.4%), Clostridiales (5.9%), Flavobacteriaceae (4.8%), Rhizobiales (4.2%), Rhodospirillaceae (3.7%), and Chromatiales (3.7%) (Table S1). Wilcoxon rank-sum test and Metastat test at family level showed that Rhizobiales, Rhodospirillaceae, and Chromatiales were differentially more abundant in the diseased tissues than the apparently healthy sections of SGA-affected *P. carnosus* colonies (*p* < 0.05, Table S1).

**Table 2:** **Summary of SIMPER analysis results for the major phyla**.

**Taxon**	**Dissim**.	**Con. %**	**Cum. %**	**Diseased**	**Healthy**	***p_t_***	***p_h_***	***p*_*M*_**
*Proteobacteria*	12.99	37.56	37.56	0.677	0.432			^*^
*Firmicutes*	5.765	16.67	54.23	0.00953	0.125		^*^	
*Bacteroidetes*	4.618	13.36	67.59	0.141	0.207			
*Cyanobacteria*	1.758	5.084	72.67	0.0114	0.0461			
*Actinobacteria*	1.646	4.761	77.43	0.0288	0.0392			
*Chlorobi*	1.425	4.120	81.55	0.0131	0.0278			
Unknown bacteria	1.422	4.111	85.66	0.0433	0.0429			
***Acidobacteria***	**1.113**	**3.220**	**88.88**	**0.0253**	**0.00307**	^*^		^*^
*Chloroflexi*	1.029	2.975	91.86	0.0228	0.0327			
***Spirochaetes***	**0.6853**	**1.982**	**93.84**	**0.00140**	**0.0151**		^*^	^*^
*Caldiserica*	0.6254	1.809	95.65	0	0.0125			
*Planctomycetes*	0.3506	1.014	96.66	0.00732	0.00444			
*SBR1093*	0.2684	0.7761	97.44	0.00580	0.00336			
***Gemmatimonadetes***	**0.2017**	**0.5833**	**98.02**	**0.00466**	**0.000626**	^*^		^*^
*TM6*	0.08939	0.2585	98.28	0.00185	7.52E-05	^*^		
*TM7*	0.08216	0.2376	98.52	0.00040	0.00164			
*Deinococcus-Thermus*	0.07275	0.2104	98.73	0.00185	0.000640			
*Verrucomicrobia*	0.06050	0.1750	98.90	0.00139	0.000545			

Dissim., Bray-Curtis Dissimilarity values; Con. %, percentage of the contributed difference; Cum. %, Cumulative percentage; Diseased, relative abundance of each bacterial taxa obtained from P. carnosus diseased tissues; Healthy, relative abundance of each bacterial taxa obtained from P. carnosus apparently healthy tissues; p_t_, p-value for the alternative hypothesis of higher abundance in diseased tissue samples by Wilcoxon rank-sum test; p_h_, p-value for the alternative hypothesis of higher abundance in apparently healthy tissue samples by Wilcoxon rank-sum test; p_M_, p-value for the alternative hypothesis by Metastats test; asterisk

* indicates significant difference between the diseased and the apparently healthy tissue samples with Wilcoxon rank-sum test or Metastats test (p < 0.05). Text in bold indicates phyla with significant statistical differences in both tests.

### Identities of bacteria exclusively associated with the diseased tissues of SGA-affected *P. carnosus*

A total of 788 OTUs were identified by 16S ribosomal RNA gene sequencing as exclusively associated with the diseased tissues and were absent in the apparently healthy tissues of SGA-affected colonies (Figure 2A). Of these 788 OTUs, 42 were consistently detected in all four replicates of the diseased condition (Table 3). BLAST analysis indicated that all 42 OTUs were of marine origin, and there were no evidence of bacteria of human or terrestrial origin (Table 3). Twenty-one of the 42 OTUs (50.0%) were identified as members of the class Alphaproteobacteria. Other bacterial taxa represented included Gammaproteobacteria (21.4% of OTUs), Bacteroidetes (9.5%), Actinobacteria (4.8%), Cyanobacteria (4.8%), Deltaproteobacteria (4.8%), and Acidobacteria (2.4%). The phylum Proteobacteria represented 76.2% of the sequenced OTUs found exclusively associated with the diseased tissues (Table 3). Of the analyzed OTUs, 2.4% had no close relatives in the NCBI database and could only be classified as unknown bacterial clones.

**Table 3:** **OTUs exclusively associated with *P. carnosus* diseased tissues**.

**OTU**	**#**	**RDP 16S rRNA reference sequences database v9**				**Closest Blastn match with NCBI GenBank *nt* database**		
		**Phylum**	**Class**	**Order**	**RDP classified taxon (80% confidence threshold)**	**Accession no**.	**% Similarity**	**Source**
763	36	*Acidobacteria*	*Acidobacteria*	*Acidobacteria*	*Acidobacteria* (Genus *Gp10*)	FJ205217	91	Uncultured Acidobacteria bacterium (hydrothermal field sediment)
783	6	*Actinobacteria*	*Actinobacteria*	*Acidimicrobiales*	*Acidimicrobineae* (unclassified)	KF886137	97	Uncultured bacterium (hydrothermal field sediment)
336	34	*Actinobacteria*	Unclassified	Unclassified	Unclassified Actinobacteria	EU491785	95	Uncultured bacterium (Ocean crust)
1385	101	*Bacteroidetes*	*Cytophagia*	*Cytophagales*	*Flammeovirgaceae* (unclassified)	GU369880	99	Uncultured Bacteroidetes bacterium (marine microbial mat)
**2093**	**6**	***Bacteroidetes***	***Flavobacteria***	***Flavobacteriales***	***Flavobacteriaceae*** **(unclassified)**	**GQ412799**	**98**	**Uncultured bacterium (coral *Porites cylindrica*)**
3513	62	*Bacteroidetes*	*Flavobacteria*	*Flavobacteriales*	*Flavobacteriaceae* (Genus *Algibacter*)	JN830951	98	Uncultured bacterium (seafloor sediment)
2567	22	*Bacteroidetes*	*Flavobacteria*	*Flavobacteriales*	*Flavobacteriaceae* (Genus *Maritimimonas*)	JX527388	95	Uncultured Flavobacteriaceae bacterium (seawater)
2382	5	*Cyanobacteria*	*Cyanobacteria*	*Cyanobacteria*	*Family IV* (Genus *GpIV*)	JQ727114	99	Uncultured bacterium (marine biofilm)
2705	24	*Cyanobacteria*	*Cyanobacteria*	*Cyanobacteria*	*Family II* (Genus *GpIIa*)	GU061669	100	Uncultured bacterium (marine sediment)
3175	36	*Proteobacteria*	*Alphaproteobacteria*	*Rhizobiales*	*Hyphomicrobiaceae* (Genus *Filomicrobium*)	HQ601714	99	Uncultured bacterium (marine biofilm)
**1986**	**48**	***Proteobacteria***	***Alphaproteobacteria***	***Rhizobiales***	***Phyllobacteriaceae* (Genus *Hoeflea*)**	**EF123312**	**99**	**Uncultured alphaproteobacterium (black band disease in coral *Siderastrea siderea*)**
1071	263	*Proteobacteria*	*Alphaproteobacteria*	*Rhizobiales*	*Phyllobacteriaceae* (unclassified)	JF272168	98	Uncultured bacterium (marine biofilm)
1706	16	*Proteobacteria*	*Alphaproteobacteria*	*Rhizobiales*	*Rhizobiales incertae sedis* (Genus *Bauldia*)	NR117251	99	*Bauldia litoralis* strain
2601	55	*Proteobacteria*	*Alphaproteobacteria*	*Rhizobiales*	*Rhizobiales* (unclassified)	JF268389	100	Uncultured bacterium (deep-sea sediment)
3389	24	*Proteobacteria*	*Alphaproteobacteria*	*Rhizobiales*	*Rhodobiaceae* (Genus *Anderseniella*)	EU346403	100	Marine sponge bacterium (*Haliclona (gellius)* sp.)
**3528**	**29**	***Proteobacteria***	***Alphaproteobacteria***	***Rhizobiales***	***Rhodobiaceae* (Genus *Anderseniella*)**	**FJ930408**	**99**	**Uncultured bacterium (coral-associated bacterial community)**
398	34	*Proteobacteria*	*Alphaproteobacteria*	*Rhodobacterales*	*Rhodobacteraceae* (Genus *Roseovarius*)	NR044424	99	*Roseovarius aestuarii* sp. from tidal flat
1763	1531	*Proteobacteria*	*Alphaproteobacteria*	*Rhodobacterales*	*Rhodobacteraceae* (Genus *Roseovarius*)	HQ242427	99	Uncultured Rhodobacteraceae bacterium (seawater)
**2586**	**22**	***Proteobacteria***	***Alphaproteobacteria***	***Rhodobacterales***	***Rhodobacteraceae* (Genus *Roseovarius*)**	**EF123384**	**99**	**Uncultured alphaproteobacterium (black band disease in coral *Siderastrea siderea*)**
**3330**	**448**	***Proteobacteria***	***Alphaproteobacteria***	***Rhodobacterales***	***Rhodobacteraceae* (Genus *Ruegeria*)**	**KF179946**	**99**	**Uncultured bacterium (white patch syndrome in coral *Porites lutea*)**
**3536**	**47**	***Proteobacteria***	***Alphaproteobacteria***	***Rhodobacterales***	***Rhodobacteraceae* (unclassified)**	**DQ200606**	**97**	**Uncultured alphaproteobacterium (coral *Montastraea annularis*)**
**3086**	**35**	***Proteobacteria***	***Alphaproteobacteria***	***Rhodobacterales***	***Rhodobacteraceae* (unclassified)**	**JF835684**	**99**	**Uncultured bacterium (bleached colonies of coral *Siderastrea stellata*)**
2625	1138	*Proteobacteria*	*Alphaproteobacteria*	*Rhodobacterales*	*Rhodobacteraceae* (unclassified)	JX405520	98	Uncultured marine bacterium (seawater)
2816	92	*Proteobacteria*	*Alphaproteobacteria*	*Rhodobacterales*	*Rhodobacteraceae* (unclassified)	GU066430	100	Uncultured bacterium (marine biofilm)
497	27	*Proteobacteria*	*Alphaproteobacteria*	*Rhodobacterales*	*Rhodobacteraceae* (unclassified)	AB694457	98	Uncultured bacterium (deep-sea sediment, depth 7111 m)
**1468**	**119**	***Proteobacteria***	***Alphaproteobacteria***	***Rhodospirillales***	***Rhodospirillaceae* (Genus *Pelagibius*)**	**GU118834**	**99**	**Uncultured bacterium (coral-associated bacterial community)**
**1644**	**107**	***Proteobacteria***	***Alphaproteobacteria***	***Rhodospirillales***	***Rhodospirillaceae* (unclassified)**	**JQ516560**	**99**	**Uncultured Rhodospirillaceae bacterium (coral *Montastraea faveolata*)**
3098	52	*Proteobacteria*	*Alphaproteobacteria*	*Sphingomonadales*	*Sphingomonadales* (unclassified)	EF491353	99	Uncultured alphaproteobacterium (seawater)
1880	16	*Proteobacteria*	*Alphaproteobacteria*	Unclassified	Unclassified Alphaproteobacteria	HQ153947	99	Uncultured bacterium (marine microbial mat)
**2325**	**357**	***Proteobacteria***	***Alphaproteobacteria***	**Unclassified**	**Unclassified Alphaproteobacteria**	**FJ202551**	**99**	**Uncultured bacterium (white plague disease in coral *Montastraea faveolata*)**
2641	24	*Proteobacteria*	*Deltaproteobacteria*	*Myxococcales*	*Myxococcales* (unclassified)	JQ580213	95	Uncultured deltaproteobacterium (oil-polluted subtital sediment)
3476	38	*Proteobacteria*	*Deltaproteobacteria*	Unclassified	Unclassified Deltaproteobacteria	AY592337	96	Uncultured bacterium (Deep-sea mud volcano)
**588**	**64**	***Proteobacteria***	***Gammaproteobacteria***	***Incertae sedis***	***Gammaproteobacteria incertae sedi*s (unclassified)**	**GU319278**	**98**	**Uncultured marine bacterium (coral *Acropora eurystoma*)**
**1277**	**11**	***Proteobacteria***	***Gammaproteobacteria***	***Incertae sedis***	***Gammaproteobacteria incertae sedis* (unclassified)**	**FJ203535**	**99**	**Uncultured bacterium (white plague disease in coral *Montastraea faveolata*)**
2513	475	*Proteobacteria*	*Gammaproteobacteria*	*Incertae sedis*	*Gammaproteobacteria incertae sedis* (Genus *Arenicella*)	EU290222	97	Uncultured bacterium (marine sponge *Tethya californiana*)
**1891**	**261**	***Proteobacteria***	***Gammaproteobacteria***	**Unclassified**	**Unclassified Gammaproteobacteria**	**FJ202524**	**99**	**Uncultured bacterium (white plague disease in coral *Montastraea faveolata*)**
1917	8	*Proteobacteria*	*Gammaproteobacteria*	Unclassified	Unclassified Gammaproteobacteria	JF261866	98	Uncultured bacterium (marine biofilm)
3469	13	*Proteobacteria*	*Gammaproteobacteria*	Unclassified	Unclassified Gammaproteobacteria	AB694447	98	Uncultured bacterium (seafloor sediment)
**689**	**160**	***Proteobacteria***	***Gammaproteobacteria***	**Unclassified**	**Unclassified Gammaproteobacteria**	**GU118018**	**99**	**Uncultured bacterium (coral-associated bacteria community)**
**1015**	**24**	***Proteobacteria***	***Gammaproteobacteria***	**Unclassified**	**Unclassified Gammaproteobacteria**	**GQ412875**	**99**	**Uncultured bacterium (coral-associated bacterial community)**
**1495**	**73**	***Proteobacteria***	***Gammaproteobacteria***	**Unclassified**	**Unclassified Gammaproteobacteria**	**AM911359**	**97**	**Uncultured bacterium (coral *Lophelia pertusa*)**
527	51	Unclassified	Unclassified	Unclassified	Unclassified Bacteria	EF629794	98	Uncultured deltaproteobacterium (sponge *Ircinia strobilina*)

These 42 OTUs were observed in all four replicates of coral diseased tissues but not in any apparently healthy tissues of SGA-affected colonies. Taxonomic classification of each OTU is given as far as it could be determined at the 80% confidence threshold with the RDP classifier based on the 16S rRNA reference sequences database v9. The closest Blastn match of each OTU in the NCBI GenBank nt database is given with the accession number, similarity and source. OTU refers to the unique identity number assigned to each OTU and # indicates the number of sequences represented by that OTU. Text in bold indicate OTUs that affiliate with sequences from other coral-associated bacteria.

The Alphaproteobacteria exhibited high diversity and represented 21 (50%) OTUs and 75.0% of total sequences (4496 of 5994 sequences) identified as exclusively associated with diseased tissues. The class was dominated by members of the Rhodobacterales (42.9% of OTUs and 56.3% of sequences among Alphaproteobacteria), followed by Rhizobiales (33.3 and 7.9%), Rhodospirillales (9.5 and 3.8%), and Sphingomonadales (4.8 and 0.9%). Unclassified alphaproteobacterial sequences constituted 9.5% of OTUs and 6.2% of all analyzed sequences obtained from the diseased tissues. Twenty-two sequences of OTU#2586 and 448 sequences of OTU#3330 exhibited high levels of sequence similarity to an uncultured bacterium (accession no. EF123384; sequence identity 99% and KF179946; 99%, respectively) in the genus *Roseovarius* associated with black band disease, and *Ruegeria* species associated with white patch syndrome (Table 3). Members of *Roseovarius* (order Rhodobacterales) were the most numerically dominant genus (26.5%) among all sequences detected as exclusively associated with the diseased tissues. Also of interest are 35 sequences of OTU#3086, which were closely related to an uncultured bacterium (JF835684; sequence identity 99%) associated with bleached colonies in the coral *Siderastrea stellata*. All remaining OTUs belonging to Rhodobacterales were closely related to the uncultured bacterium (DQ200606; sequence identity 97%, GU066430; 100%, and AB694457; 98%, respectively) isolated from the coral *Montastraea annularis*, marine biofilm, and deep-sea sediment, respectively.

Wilcoxon rank-sum test and Metastats test identified a number of bacterial taxa which were significantly more abundant in the diseased tissues than in the apparently healthy tissues of SGA-affected colonies. The most noteworthy of these were OTUs belonging to the order Rhizobiales and Rhodospirillales in the class Alphaproteobacteria. Of particular interest are 48 sequences of OTU#1986 which exhibited high levels of sequence similarity to *Hoeflea* species associated with black band disease in the coral *Siderastrea siderea*. Also of interest are 119 sequences of OTU#1468, and 107 sequences of OTU#1644, which were closely related to Rhodospirillaceae species (GU118834, sequence identity 99% and JQ516560; 99%) isolated from the bacterial community of threatened Caribbean corals in the genus *Pelagibius*, and the coral *Montastraea faveolata*, respectively. Two OTUs (OTU#1880 and OTU#2325) with 99% sequence identity within Alphaproteobacteria were designated unclassified by the RDP (Table 3). BLAST analysis indicated that 357 sequences of OTU#2325 were closely related to an uncultured bacterium (FJ202551; sequence identity 99%) associated with white plague disease in the Caribbean coral *Montastraea faveolata*.

The class Gammaproteobacteria represented 9 (21.4%) OTUs and 18.2% of total sequences (1089 of 5647 sequences) identified as exclusively associated with diseased tissues (Table 3). Six OTUs (OTU#1891, OTU#1917, OTU#3469, OTU#689, OTU#1015, and OTU#1495) with high sequence identity (≥97%) represented an organism that was unrelated to described families within the Gammaproteobacteria and was designated unclassified by the RDP. Other OTUs identified (OTU#588, OTU#1277, and OTU#2513) belonged to the uncertain status of *incertae sedis* of the Gammaproteobacteria. OTU#2513 was the only OTU assigned to the genus *Arenicella*, while all other OTUs were taxonomically unclassified at the species level. Both OTU#1277 and OTU#1891 were closely related to species (FJ203434; sequence identity 99% and FJ202524; 99%) found associated with white plague disease in corals.

All the remaining OTUs isolated from the diseased tissues and absent from the apparently healthy tissues belonged to the phyla Bacteroidetes, Actinobacteria, Cyanobacteria, and Acidobacteria (Table 3). The most noteworthy of these was OTU#2093, which belonged to the family Flavobacteriaceae in Bacteroidetes. BLAST analysis indicated that this OTU was closely related to an uncultured bacterium (GQ412799; sequence identity 98%) from the coral-associated bacterial communities of *Porites cylindrica*. All of these OTUs were present only in samples of the diseased tissues and absent from samples of the apparently healthy tissues of SGA-affected colonies.

## Discussion

### Bacterial diversity of the apparently healthy and the diseased tissues of *P. carnosus*

Coral-associated bacterial communities form complex interactions that vary with species, geographical location, seasons, and health states (Kvennefors et al., 2010; Ceh et al., 2011; Morrow et al., 2012; Roder et al., 2014; McKew et al., 2015; Zhang et al., 2015). Several investigators have shown that the diversity of the bacterial communities associated with identical coral species varied with geographic location and seasonal changes (Morrow et al., 2012; Carlos et al., 2013; McKew et al., 2015; Pantos et al., 2015; Zhang et al., 2015). For instance, a recent study has demonstrated that the bacterial communities associated with *Acropora* and *Porites* corals were more diverse in the Mexican Caribbean than their Indonesian counterparts (McKew et al., 2015), suggesting that coral-associated bacterial communities are highly dynamic and diverse. In this study, we have observed a diverse suite of bacterial species from a number of phyla, including Proteobacteria, Bacteroidetes, Cyanobacteria, and Actinobacteria. Members of these phyla have also been reported to be associated with corals of other species (Cárdenas et al., 2011; Garcia et al., 2013; Séré et al., 2013; Kellogg et al., 2014; Meyer et al., 2014; Fernando et al., 2015). It is noted that the sample size in this study was limited due to the conservation value of the coral (replicate number = 4), and this may have led to underestimating the overall diversity of the bacterial communities.

Alphaproteobacteria was identified as the predominant class, representing almost twice the number of sequences compared to the Gammaproteobacteria. These findings contradict with the results of our previous work, which have revealed dominance of gammaproteobacterial sequences among remote healthy and diseased *P. caronsus* colonies, in particular *Vibrio* species (Chiu et al., 2012). Analysis of 16S rRNA gene sequences indicated that the Gammaproteobacteria represented the second most dominant group, with Vibrionaceae representing only 0.02% of the analyzed sequences in the diseased tissues. The difference in dominance is suggested to be related to the use of culture-independent or culture-dependent approaches for identifying bacterial communities. Consistent with our results, studies based on culture-independent methods using direct amplification and sequencing in coral tissues have observed dominance of alphaproteobacterial sequences (Pantos et al., 2003; Godwin et al., 2012). On the contrary, culture-dependent studies have detected gammaproteobacterial sequences as the dominant cultivable group in reef-building corals (Pantos et al., 2003; Chiu et al., 2012; Godwin et al., 2012). The dominance of this group in culturing techniques, particularly members of the order Vibrionales, is suggested to be associated to the groups' resilience to antibiotics and their ability to produce antibiotics that inhibit the proliferation of other bacteria (Long and Azam, 2001).

Diversity and richness of the bacterial communities associated with the apparently healthy and the diseased tissues of SGA-affected colonies were comparable at the selected number of sequences, indicating similar diversity and richness of the bacterial communities. These results are inconsistent with findings which have reported higher bacterial diversity in the diseased tissues than the apparently healthy tissues of corals affected by diseases, such as white plague disease (Pantos et al., 2003) and yellow band disease (Cróquer et al., 2012; Closek et al., 2014). However, comparison of the bacterial community composition between the two conditions using Bray-Curtis similarity revealed clear differences at the OTU level.

### Differentially abundant bacterial taxa in apparently healthy vs. diseased tissues

Our statistical analysis indicated that the largest contributor to the difference in abundance between the apparently healthy and the diseased tissues was Proteobacteria. This result is consistent with previously reported observations of bacterial communities associated with black band disease (Sato et al., 2010; Miller and Richardson, 2011), white plague disease (Sunagawa et al., 2009; Cárdenas et al., 2011), and white patch syndrome (Séré et al., 2013). Members of the Acidobacteria, Rhizobiales, and Rhodospirillaceae were more abundant in the diseased tissues, as have been shown previously in bleached corals (Mouchka et al., 2010) and in corals with white plague disease (Cárdenas et al., 2011; Roder et al., 2014). Furthermore, we identified Rhodobacteraceae as the prominent family contributing to the most difference in abundance between the apparently healthy and the diseased tissues. Our results well relate with studies which have observed Rhodobacteraceae contributing as much as 75% of the difference between healthy and diseased corals (Roder et al., 2014).

### Bacterial groups strongly associated with SGA in *P. carnosus*

There were a number of OTUs that were strongly associated with the diseased tissues and have a high level of 16S rRNA gene sequence similarity to bacteria associated with marine diseases or known pathogens. Most OTUs with high sequence similarity to bacteria previously isolated from marine diseases were classified as belonging to the Rhodobacteraceae, Rhizobiales, Gammaproteobacteria, and Cytophaga-Flavobacterium-Bacteroidetes (CFB).

Within the Rhodobacteraceae, all OTUs that could be classified to the genus level (4 out of 9) belong to the *Roseobacter* clade, whose members are closely related to bacteria implicated in other marine diseases. Of particular importance is OTU#2586, which exhibited high levels of sequence similarity to an alphaproteobacterium associated with black band disease in the coral *Siderastrea siderea* (Sekar et al., 2008). OTU#2586 belong to the genus *Roseovarius*, which contains the known pathogen *Roseovarius crassostreae*, the causative agent associated with Juvenile Oyster Disease (JOD) in the cultured Eastern oyster *Crassostrea virginica* (Boettcher et al., 2005; Sekar et al., 2006, 2008). It is worthwhile noting that the relative abundance of *Roseovarius* makes up 25.6% of all sequences identified in the diseased tissues, but is absent in the apparently healthy tissue. Furthermore, OTU#3330 represented a bacterium assigned to the genus *Ruegeria* found in association with white patch syndrome in the coral *Porites lutea* (Séré et al., 2013). Members of the *Ruegeria* have previously been reported to be associated with yellow band disease in Fungiidae corals (Apprill et al., 2013). Moreover, the bacterium *Ruegeria atlantica* has been shown to be associated with shellfish poisoning by producing compounds that lyse the toxic dinoflagellate *Alexandrium catenella* (Amaro et al., 2005). *Roseobacter* species are known to contain phenotypes for the production of antibacterial compounds (Bruhn et al., 2007), which may have caused disruption of the native commensal coral bacterial community.

Within the Rhizobiales, OTU#1986 was assigned to the genus *Hoeflea* representing an uncultured bacterium found in association with corals affected by black band disease (Sekar et al., 2008). Members of the *Hoeflea* have previously been reported to be associated with marine diseases (Palacios et al., 2006; Fiebig et al., 2013). For example, *Hoeflea alexandrii*, which was isolated from the toxin-producing dinoflagellate *Alexandrium minutum*, was found to be associated with paralytic shellfish poisoning events (Palacios et al., 2006). In addition, *Hoeflea phototrophica* have previously been identified to be associated with the toxic dinoflagellate *Prorocentrum lima* (Fiebig et al., 2013). The Rhizobiales also contains many known pathogens, such as *Aurantimonas coralicida*, which causes the white plague type II disease in the coral *Dichocoenia stokesi* (Denner et al., 2003), and *Brucella* spp., which is responsible for brucellosis in a wide range of marine vertebrates (Carvalho et al., 2010).

Within the Gammaproteobacteria, OTU#1277 and OTU#1891 were found closely related to the uncultured bacterium associated with the coral *Montastraea faveolata* affected by white plague disease (Sunagawa et al., 2009). It is worthwhile noting that we did not detect any OTUs belonging to Vibionaceae in the bacterial community exclusively associated with the diseased tissues. The pathogenicity of *Vibrio* spp. in coral diseases is well documented. For instance, *V. coralliilyticus* and *V. harveyi* have been attributed for white syndrome and white band disease (Sussman et al., 2008; Luna et al., 2010; Sweet et al., 2014), and *V. shiloi* has been found in association with bleaching in the coral species *Oculina patagonica* (Kushmaro et al., 2006). Many studies have detected *Vibrio* spp. in both healthy and diseased corals (Breitbart et al., 2005; Chiu et al., 2012; Wilson et al., 2012). Yet the role of Vibrionaceae in coral disease remains unclear, as is the role of other species within the Gammaproteobacteria in the development of SGA.

All remaining OTUs were assigned to the Flammeovirgaceae and Flavobacteriaceae. These taxa belong to the CFB group, which have been implicated in marine diseases (Pinhassi et al., 1997; Romero et al., 2010). For example, *Cytophaga* species have previously been proposed to be the causative agents of black band disease (Miller and Richardson, 2011), and Flavobacteriaceae species have been found in association with marine eukaryote diseases (Chistoserdov et al., 2012; Quinn et al., 2012). The CFB group also contains a number of known bacterial pathogens, including *Tenacibaculum maritimum*, which is responsible for the fish disease tenacibaculosis in turbot (Romero et al., 2010; Faílde et al., 2013). In many marine ecosystems, CFB members are known to be important for the turnover of organic matter (Kirchman, 2002), which may contribute to success in colonization of CFB bacteria via the production of rich organic habitats. Additionally, the group contains most of the algicidal bacteria isolated from marine environments (Mayali and Azam, 2004; Tian et al., 2012). Members possessing algicial properties have been proposed to play a role in the causation of Yellow Blotch Disease (Cervino et al., 2004), and the rapid progression of tissue whitening in white plague disease in corals (Sunagawa et al., 2009).

These bacterial groups strongly associated with the diseased coral tissues may contain potential pathogens involved in the development of SGA in *P. carnosus*. Nevertheless, it is possible that the corals' defense capabilities may be compromised at the diseased status, allowing the colonization and proliferation of competitive bacteria (Pantos et al., 2003; Cárdenas et al., 2011; Godwin et al., 2012). Future work should focus on enriching isolates from these groups so that they may be used in infection experiments to attempt to determine the bacterial pathogen(s) of SGA.

## Conclusion

We established that SGA in *P. carnosus* produces significant differences in bacterial community composition between the apparently healthy and the diseased tissues at OTU level. We also report differentially abundant members between the two conditions in Acidobacteria, Rhizobiales and Rhodospirillaceae. There were certain specific OTUs within the Rhodobacteraceae, Rhizobiales, Gammaproteobacteria, and CFB that found to be exclusively associated with the diseased tissues, which may contain potential pathogens or opportunistic colonizers that proliferate during the development of SGA. Nevertheless, our one-time sampling snapshot data makes it impossible to determine whether OTUs in these groups are primary pathogens or bacteria fueled by metabolic by-products of the disease. Since the samples were only obtained from SGA-affected colonies, we were also not able to determine whether distinct differences are present between the bacterial communities of tissues from healthy corals and apparently healthy tissues from diseased corals. The corals may have been infected and already progressed into a diseased state prior to the appearance of any visible signs. Future studies based on experimental inoculation and testing of Koch's postulates are needed in order to derive a conclusion about the role of these bacteria in the pathogenesis and the etiology of the SGA.

### Conflict of interest statement

The authors declare that the research was conducted in the absence of any commercial or financial relationships that could be construed as a potential conflict of interest.
