# Supplementary material for: Pyrosequencing of the bacteria associated with Platygyra carnosus corals with skeletal growth anomalies reveals differences in bacterial community composition in apparently healthy and diseased tissues
Source: Front Microbiol. 2015 Oct 20;6:1142. doi: 10.3389/fmicb.2015.01142 (PMC4611154; doi:10.3389/fmicb.2015.01142)
Supplement: Supplementary file 1 [file DataSheet1.DOCX]

**Supplementary Material**

**Table S1 Summary of SIMPER analysis results at the family level.** Dissim. = Bray-Curtis Dissimilarity values; Con. % = percentage of the contributed difference; Cum. % = Cumulative percentage; Diseased = relative abundance of each bacterial taxa obtained from *P. carnosus* diseased tissues; Healthy = relative abundance of each bacterial taxa obtained from *P. carnosus* apparently healthy tissues; *p_t_* = *p* value for the alternative hypothesis of higher abundance in diseased tissue samples by Wilcoxon rank-sum test; *p_h_* = *p* value for the alternative hypothesis of higher abundance in apparently healthy tissue samples by Wilcoxon rank-sum test; *p_M_* = *p* value for the alternative hypothesis by Metastats test; asterisk * indicates significant difference between the diseased and the apparently healthy tissue samples with Wilcoxon rank-sum test or Metastats test (*p* < 0.05). Text in bold indicates taxa with significant statistical differences in both tests.

| Taxon | Dissim. | | Con. % | | Cum.% | Diseased | Healthy | *P_t_* | *P_h_* | *P_M_* |
| --- | --- | --- | --- | --- | --- | --- | --- | --- | --- | --- |
| Rhodobacteraceae | 9.19 | 14.56 | | 14.56 | | 0.258 | 0.126 |  |  |  |
| Flammeovirgaceae | 4.01 | 6.35 | | 20.9 | | 0.016 | 0.094 |  | * |  |
| Clostridiales | 3.75 | 5.93 | | 26.83 | | 0.002 | 0.077 |  | * |  |
| Flavobacteriaceae | 3.05 | 4.83 | | 31.67 | | 0.077 | 0.072 |  |  |  |
| Rhizobiales | **2.63** | **4.17** | | **35.83** | | **0.063** | **0.011** | ***** |  | ***** |
| Rhodospirillaceae | **2.34** | **3.71** | | **39.54** | | **0.074** | **0.03** | ***** |  | ***** |
| Chromatiales | **2.34** | **3.7** | | **43.24** | | **0.058** | **0.011** | ***** |  | ***** |
| Sinobacteraceae | 1.64 | 2.6 | | 45.84 | | 0.03 | 0.048 |  |  |  |
| Unknown Bacteria | 1.42 | 2.25 | | 48.09 | | 0.043 | 0.043 |  |  |  |
| Chloroflexales | 1.28 | 2.02 | | 50.11 | | 0.001 | 0.026 |  |  |  |
| Chlorobiaceae | 1.17 | 1.85 | | 51.96 | | 0.012 | 0.019 |  |  |  |
| Phyllobacteriaceae | 1.06 | 1.68 | | 53.65 | | 0.021 | 0.032 |  |  |  |
| Dehalobacteriaceae | 1.05 | 1.67 | | 55.31 | | 0.003 | 0.02 |  |  |  |
| Enterobacteriaceae | 0.99 | 1.57 | | 56.88 | | 0 | 0.02 |  |  |  |
| Cyanobacteria_CAB-I | 0.95 | 1.5 | | 58.38 | | 0.002 | 0.02 |  |  | * |
| Unknown Gammaproteobacteria | 0.87 | 1.38 | | 59.76 | | 0.024 | 0.014 |  |  |  |
| Actinobacteria koll13 | 0.87 | 1.38 | | 61.14 | | 0.018 | 0.005 |  |  |  |
| Unknown Gammaproteobacteria | 0.84 | 1.33 | | 62.47 | | 0.022 | 0.008 |  |  |  |
| Unknown Alphaproteobacteria | 0.81 | 1.28 | | 63.75 | | 0.008 | 0.014 |  |  |  |
| Streptophyta | 0.77 | 1.22 | | 64.97 | | 0 | 0.015 |  |  |  |
| Unknown Bacteroidetes | 0.69 | 1.09 | | 66.06 | | 0.017 | 0.005 |  |  | * |
| Saprospiraceae | 0.68 | 1.08 | | 67.14 | | 0.019 | 0.009 |  |  |  |
| Myxococcales | 0.68 | 1.08 | | 68.21 | | 0.015 | 0.002 |  |  | * |
| Caldiserica_062DZ04 | 0.63 | 0.99 | | 69.20 | | 0.000 | 0.013 |  |  |  |
| Desulfobacteraceae | 0.57 | 0.90 | | 70.10 | | 0.001 | 0.012 |  |  | * |
| Bacteroidales | **0.54** | **0.86** | | **70.96** | | **0.000** | **0.011** |  | ***** | ***** |
| Acidobacteria_Sva0725 | **0.52** | **0.83** | | **71.79** | | **0.011** | **0.001** | ***** |  | ***** |
| Acidimicrobiales_JdFBGBact | 0.45 | 0.71 | | 72.50 | | 0.002 | 0.008 |  |  |  |
| Spirochaetaceae | 0.44 | 0.70 | | 73.20 | | 0.000 | 0.009 |  |  | * |
| Halanaerobiaceae | 0.42 | 0.66 | | 73.86 | | 0.001 | 0.008 |  |  |  |
| Moraxellaceae | 0.40 | 0.63 | | 74.49 | | 0.002 | 0.007 |  |  |  |
| Unknown Chlorobi | 0.38 | 0.61 | | 75.09 | | 0.000 | 0.008 |  |  |  |
| Oceanospirillales_HTCC2089 | 0.36 | 0.57 | | 75.67 | | 0.008 | 0.001 | * |  |  |
| Acidimicrobiales_CL500-29 | 0.36 | 0.57 | | 76.23 | | 0.003 | 0.007 |  |  |  |
| Rhodobacterales | 0.36 | 0.56 | | 76.79 | | 0.008 | 0.000 | * |  |  |
| Synechococcaceae | 0.35 | 0.56 | | 77.35 | | 0.002 | 0.008 |  |  |  |
| Solibacteraceae | 0.34 | 0.54 | | 77.90 | | 0.007 | 0.000 |  |  |  |
| Deltaproteobacteria_NB1-j | 0.32 | 0.51 | | 78.41 | | 0.007 | 0.001 | * |  |  |
| Burkholderiaceae | 0.32 | 0.51 | | 78.92 | | 0.000 | 0.006 |  |  |  |
| Anaerolineae_A4b | **0.31** | **0.49** | | **79.42** | | **0.007** | **0.000** | ***** |  | ***** |
| Sphingomonadaceae | 0.31 | 0.49 | | 79.90 | | 0.002 | 0.007 |  |  |  |
| Rhodothermaceae | 0.31 | 0.48 | | 80.39 | | 0.007 | 0.002 |  |  |  |
| Rhizobiales | **0.30** | **0.47** | | **80.86** | | **0.006** | **0.000** | ***** |  | ***** |
| Unknown Deltaproteobacteria | 0.27 | 0.43 | | 81.28 | | 0.007 | 0.003 |  |  |  |
| Methylophilaceae | 0.26 | 0.41 | | 81.70 | | 0.005 | 0.000 | * |  |  |
| Sphingomonadaceae | 0.25 | 0.39 | | 82.09 | | 0.000 | 0.005 |  |  |  |
| Endozoicimonaceae | 0.24 | 0.38 | | 82.47 | | 0.000 | 0.005 |  |  |  |
| Rhizobiaceae | 0.24 | 0.38 | | 82.85 | | 0.000 | 0.005 |  |  |  |
| Sphingomonadales | 0.24 | 0.38 | | 83.22 | | 0.005 | 0.005 |  |  |  |
| Pseudomonadaceae | 0.23 | 0.37 | | 83.59 | | 0.000 | 0.005 |  |  |  |
| Methylobacteriaceae | 0.23 | 0.37 | | 83.96 | | 0.000 | 0.005 |  |  |  |
| Chroococcales | 0.23 | 0.36 | | 84.33 | | 0.005 | 0.000 |  |  |  |
| Clostridiales_Family_XI | 0.21 | 0.34 | | 84.66 | | 0.000 | 0.004 |  |  |  |
| Oceanospirillales_OM60 | 0.20 | 0.32 | | 84.99 | | 0.008 | 0.007 |  |  |  |
| Spirochaetes_SHA-4 | 0.20 | 0.32 | | 85.31 | | 0.001 | 0.004 |  |  |  |
| Gemmatimonadetes | **0.20** | **0.32** | | **85.63** | | **0.005** | **0.001** | ***** |  | * |
| Sphingobacteriaceae | 0.20 | 0.31 | | 85.94 | | 0.000 | 0.004 |  |  |  |
| Bradyrhizobiaceae | 0.19 | 0.31 | | 86.25 | | 0.001 | 0.004 |  |  | * |
| Anaerolineae_GCA004 | 0.18 | 0.29 | | 86.54 | | 0.003 | 0.002 |  |  |  |
| Unknown Deltaproteobacteria | 0.18 | 0.29 | | 86.83 | | 0.001 | 0.004 |  |  |  |
| Deltaproteobacteria_NB1-j | 0.18 | 0.29 | | 87.11 | | 0.004 | 0.000 |  |  |  |
| Hahellaceae | 0.18 | 0.29 | | 87.40 | | 0.004 | 0.000 |  |  |  |
| Unknown Alphaproteobacteria | 0.18 | 0.28 | | 87.68 | | 0.004 | 0.003 |  |  |  |
| Propionibacteriaceae | 0.18 | 0.28 | | 87.96 | | 0.000 | 0.004 |  |  |  |
| Cryomorphaceae | 0.17 | 0.27 | | 88.23 | | 0.001 | 0.004 |  |  |  |
| Clostridiaceae | 0.17 | 0.27 | | 88.51 | | 0.002 | 0.003 |  |  |  |
| Actinomycetales | 0.17 | 0.27 | | 88.77 | | 0.000 | 0.003 |  |  |  |
| Staphylococcaceae | 0.16 | 0.26 | | 89.03 | | 0.000 | 0.003 |  |  |  |
| SBR1093 | 0.16 | 0.25 | | 89.29 | | 0.005 | 0.002 |  |  | * |
| Chlorothrixaceae | 0.16 | 0.25 | | 89.53 | | 0.003 | 0.000 |  |  |  |
| Vibrionaceae | 0.15 | 0.23 | | 89.77 | | 0.003 | 0.001 |  |  |  |
| Rhodobacteraceae | 0.15 | 0.23 | | 90 | | 0.001 | 0.003 |  |  |  |
| Acidimicrobiales | 0.14 | 0.22 | | 90.22 | | 0.002 | 0.002 |  |  |  |
| Catabacteriaceae | 0.14 | 0.22 | | 90.44 | | 0.000 | 0.003 |  |  |  |
| Acidimicrobiales_ntu14 | 0.13 | 0.20 | | 90.64 | | 0.001 | 0.002 |  |  |  |
| Alteromonadaceae | 0.13 | 0.20 | | 90.85 | | 0.002 | 0.001 |  |  |  |
| Chloracidobacteria | 0.13 | 0.20 | | 91.05 | | 0.003 | 0.001 |  |  |  |

**Figure S1** Relative abundance (%) of major bacterial phyla (except Proteobacteria into class) in sequenced OTUs from four apparently healthy and four diseased tissue samples. Healthy – apparently healthy tissues of SGA affected *P. carnosus* colonies, Diseased – diseased tissues of SGA affected colonies. Only OTUs identified by 16S rRNA gene sequence analysis are presented. Stacked bars were calculated from the mean of relative sequence abundance belonging to each phylum in each sample condition.

**Figure S1**

**
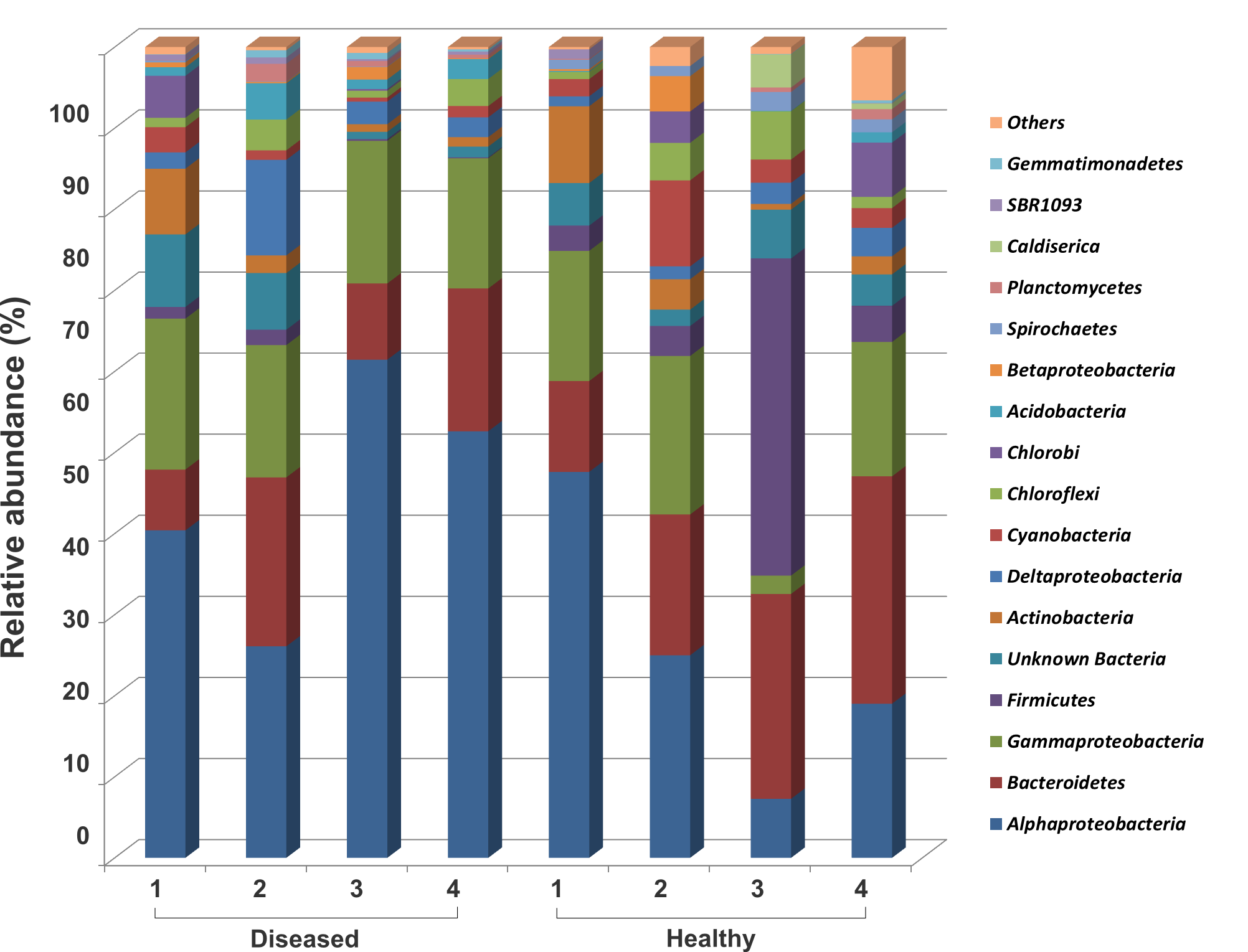
**
